# Mendelian Randomization and Transcriptome Analysis Identify Ischemic Stroke Biomarkers With Putative Relevance to Cerebrospinal Fluid

**DOI:** 10.1155/bmri/2880611

**Published:** 2026-07-08

**Authors:** Jingwei Xiong, Jie Zhang, Xuemei Cheng, Zhiqiang Zhou, Lidong Zhang, Chunlong Chen

**Affiliations:** ^1^ Department of Anesthesiology, Jinling Hospital, Affiliated Hospital of Medical School, Nanjing University, Nanjing, Jiangsu, China, nju.edu.cn

**Keywords:** biomarkers, circulating proteins, ischemic stroke, mendelian randomization, transcriptome analysis

## Abstract

**Background:**

Circulating proteins have been associated with the pathogenesis of ischemic stroke (IS), yet its biomarkers remain underutilized. Using plasma protein GWAS data with putative relevance to CSF, this study integrated mendelian randomization (MR) and transcriptomics to identify potential IS biomarkers.

**Methods:**

A two‐sample MR analysis was undertaken to determine the genetic association between circulating protein levels and IS. The identification of differentially expressed genes (DEGs) in the GSE268634 and GSE262257 datasets was carried out via the transcriptomic analysis. Candidate biomarkers overlapping MR‐derived genes (MRGs) and DEGs underwent functional enrichment, protein‐protein interaction (PPI), and machine learning (LASSO/SVM‐RFE) screening. Biomarker mechanisms were assessed via gene set enrichment analysis (GSEA), immune infiltration, and hypothesis‐generating drug prediction. The validation included RT‐qPCR and immunohistochemistry in MCAO/R rats.

**Results:**

The MR analysis identified 157 circulating protein‐related MRGs with suggestive genetic associations with IS. Transcriptomics identified 4144 DEGs, and 46 overlapping with MRGs. Functional enrichment highlighted their roles in cell adhesion and immune responses. Machine learning identified six candidate biomarkers, among which CDH7, MGAT4C, and ITPKC exhibited both high diagnostic accuracy (AUC > 0.7) and consistently differential expression, and were therefore prioritized as putative biomarkers. GSEA revealed that CDH7 and MGAT4C were positively correlated, whereas ITPKC was negatively correlated with the calcium signaling pathway. Immune infiltration analysis showed that CDH7 and MGAT4C were negative, whereas ITPKC was positively correlated with immune cells. Computationally predicted drugs including genistein and pioglitazone may alleviate IS damage, though this requires experimental confirmation. RT‐qPCR and immunohistochemistry indicated markedly high CDH7 and MGAT4C expression, whereas low ITPKC expression was in MCAO/R rats.

**Conclusion:**

CDH7, MGAT4C, and ITPKC are genetically associated and transcriptionally altered candidates derived from circulating protein‐related analyses for IS, warranting further investigation.

## 1. Background

Stroke is a leading global health concern and is broadly categorized into two main types: ischemic and hemorrhagic [1]. Among these, ischemic stroke (IS) caused by the obstruction of cerebral blood flow, accounts for the majority of cases and ranks as the second leading cause of mortality and the third major cause of long‐term disability worldwide [2]. The pathophysiology of IS involves a cascade of events following the initial ischemic insult, including neuronal death and impairments, such as deficits in motor control, cognition, mood regulation, speech, swallowing, and autonomic functions [3, 4].

The progression and severity of IS‐related damage are influenced by various molecular and cellular mechanisms, including oxidative stress, excitotoxicity, inflammation, apoptosis, and the release of neurotoxic endogenous substances [5]. Although these mechanisms have been widely studied in brain tissue and blood, the role of cerebrospinal fluid (CSF) in IS remains relatively underexplored. CSF directly bathes the brain and spinal cord, serving as a critical medium for neuronal signaling and neuroprotection. Although direct CSF protein measurement is challenging in large cohorts, plasma circulating proteins can offer indirect insights into CNS pathology [6]. Here, we leverage plasma protein GWAS data with putative relevance to CSF to identify potential IS biomarkers.

Although extensive research has investigated plasma protein biomarkers and their associations with stroke and its risk factors [7, 8], studies focusing on circulating proteins in the context of IS are limited. The regulatory mechanisms governing these proteins in IS pathogenesis remain largely unknown. To address this gap, novel analytical approaches are required. MR offers a promising strategy to assess genetic associations that are consistent with putative causal relationships between biomarkers and disease outcomes by using genetic variants as instrumental variables (IVs) [9]. The integration of MR with high‐throughput omics technologies and advanced bioinformatics has enabled deeper insights into disease etiology [10].

In this study, we employed bioinformatic analyses to identify and characterize key circulating proteins potentially involved in IS and investigated their underlying molecular mechanisms (Figure S1). Our findings aim to enhance the understanding of the protein‐mediated mechanisms in IS and contribute to the development of novel diagnostic and therapeutic strategies.

## 2. Method

### 2.1. Mendelian Randomization Analysis

#### 2.1.1. Data Sources

For exposure factors, GWAS summary statistics for circulating protein levels were obtained from the IEU OpenGWAS database (https://gwas.mrcieu.ac.uk/). A total of 3589 protein traits measured in plasma samples from European‐ancestry populations were included. Detailed information on each protein trait, including sample size, population ancestry, and measurement method, is provided in Table S1. Although these data are derived from plasma, circulating proteins can reflect central nervous system processes due to blood‐brain barrier permeability, providing a rationale for their investigation as CSF‐relevant biomarkers despite the lack of direct CSF measurements in this GWAS dataset. For the outcome variable, data for IS were retrieved from the IEU OpenGWAS database (trait ID ebi‐a‐GCST90018864). This dataset was based on the European population and included 11,929 IS cases and 472,192 controls with 24,174,314 single nucleotide polymorphisms (SNPs).

#### 2.1.2. IV Selection

To ensure the validity of IVs, we rigorously enforced the core assumptions of MR [11]:

Relevance assumption: Only SNPs significantly associated with the exposure were selected as IVs. To identify the IVs, we first selected SNPs reaching genome‐wide significance (*p* < 5 × 10^−8^) with clumping (*r* ^2^ < 0.001, distance = 10,000 kb). However, this stringent threshold resulted in a limited number of IVs for many protein traits. To capture additional potential instruments while minimizing weak instrument bias, we adopted a more liberal threshold of *p* < 5 × 10^−6^, which has been used in previous exploratory MR studies. The strength of instruments was quantified using the F‐statistic. All retained IVs exhibited F‐statistics > 10, substantially exceeding the empirical threshold to minimize weak instrument bias.

Independence assumption: SNPs were clumped (*r* ^2^ < 0.001,≥ 10,000 kb) to eliminate linkage disequilibrium (LD), ensuring genetic instruments were independent.

#### 2.1.3. Two‐Sample MR Analysis

To further validate instrument strength, the effect alleles and sizes between exposures and outcomes were evaluated using the “harmonise_data” function in the “TwoSampleMR” package. Five methods were used to perform the MR analysis of causality: MR Egger, weighted median, inverse variance weighted (IVW), simple mode, and weighted mode.

The IVW method, employing multiplicative random‐effects meta‐analysis of Wald ratios (SNP‐outcome association/SNP‐exposure association), served as the primary approach. This method assumes balanced horizontal pleiotropy (average pleiotropic effect zero). Cochran′s Q statistic assessed heterogeneity, and a random‐effects model was used to account for it, providing more conservative estimates. IVW results with *p* < 0.05 were considered statistically significant for causal inference. Odds ratios (OR) > 1 indicated risk factors; OR < 1 indicated protective factors. Results were visualized using scatter plots, funnel plots, and forest plots.

Since summary‐level GWAS data were used, missing genotypes had been imputed and quality‐controlled in the original studies. During harmonization of IVs, the harmonise_data function from the TwoSampleMR package automatically excluded SNPs with mismatched or ambiguous allele information.

#### 2.1.4. Sensitivity Analysis for MR Analysis

The MR results were analyzed using sensitivity tests, which involved heterogeneity, horizontal pleiotropy, and leave‐one‐out (LOO) analysis. For heterogeneity, Cochran′s Q test was carried out, with a *p* value exceeding 0.05 indicating an absence of marked heterogeneity. The MR pleiotropy test function was utilized for identifying horizontal pleiotropy, with *p* > 0.05 suggesting that SNPs impacted the outcome only via exposure factors. Lastly, to find out if a single SNP may significantly change the overall impact, the MR LOO function was used for the LOO analysis. These comprehensive sensitivity analyses collectively ensured the robustness of MR estimates by addressing key assumptions and potential biases.

Genes showing a genetic association with the outcome and meeting the criteria of sensitivity analysis were identified as MR‐derived genes (MRGs). To evaluate the directionality of the relationship between MR genes and IS, Steiger directional analysis was performed. A *p* value < 0.05 indicated a “true” result, suggesting that the causal direction was correct and unaffected by reverse causation. The consistency of results across all sensitivity tests reinforced the validity of genetic association inference, minimizing the risk of false‐positive findings.

### 2.2. Transcriptomic Analysis

#### 2.2.1. Data Acquisition

The transcriptomic training (GSE268634) and validation (GSE262257) datasets were both acquired from the Gene Expression Omnibus (GEO) database (https://www.ncbi.nlm.nih.gov/geo/). These datasets comprised three samples from the sham‐operated (control) rat group and the samples from the MCAO/R rat model group (cerebral ischemia‐reperfusion).

#### 2.2.2. Identification of Differentially Expressed Genes (DEGs)

The DEGs between the disease and control groups were evaluated with “DESeq2”. The selection criteria were: *p* < 0.05 and |log2 *F* *C*| > 1. Volcano plots and heat maps, generated using “ggplot2” and “heat map” in R, respectively, were utilized to visualize DEG levels. To identify the overlapping genes between DEGs and MRGs, the generation of a Venn diagram was carried out while employing the VennDiagram package in R, with the shared genes representing candidate genes. The rationale for intersecting MRGs with DEGs is based on a triangulation approach. Although MR identifies genes whose genetically predicted protein levels are associated with IS risk over the lifespan, DEGs capture acute transcriptional responses to ischemic injury. An overlapping gene provides convergent evidence from two independent biological layers (genetic susceptibility and acute pathophysiology) thereby increasing confidence in its relevance to IS. We acknowledge that this conservative approach may miss causal genes that do not show acute differential expression. However, this strategy prioritizes candidates with the highest probability of direct involvement in IS pathogenesis for downstream functional validation, reducing the risk of false positives from MR or transcriptomic analysis alone.

#### 2.2.3. Functional Enrichment Analysis and Protein‐Protein Interaction (PPI)

Gene Ontology (GO) and Kyoto Encyclopedia of Genes and Genomes (KEGG) enrichment analyses of the candidate genes was performed in clusterProfiler in R, with a threshold of *p* < 0.05. GO analysis comprises biological processes (BP), cellular components (CCs), and molecular functions (MFs). The GO and KEGG enrichment data were visualized via the GOplot package in R.

Furthermore, a PPI network was established using the STRING database (interaction score > 0.15) (https://cn.string-db.org/). Clustering was undertaken with Cytoscape, and the MCODE plugin for Molecular Complex Detection was used with parameters set as follows: degree cutoff = 2, node score cutoff = 0.2, k − core = 2, and maximum depth = 100.

#### 2.2.4. Machine Learning Methods

The candidate differential genes were subjected to the least absolute shrinkage and selection operator (LASSO) regression analysis using the glmnet in R, with the binomial family and alpha set to 1. Furthermore, the features were selected via support vector machine‐recursive feature elimination (SVM‐RFE) algorithm using the e1071 package with k‐fold cross‐validation. To identify the intersecting genes identified by the above algorithms, the VennDiagram package in R was used to create a Venn diagram. Lastly, the intersecting genes were selected as the candidate biomarkers for IS occurrence and development.

#### 2.2.5. Receiver Operating Characteristic (ROC) Curves and Correlations

The ROC curves of candidate biomarkers were generated using the pROC package in R. Additionally, the area under the curve (AUC) values were also assessed, and genes with an AUC > 0.7 in both datasets were selected as key genes for further analysis. The expression levels of key genes were assessed in the training and validation datasets using *t*‐tests. Key genes indicating significant differential expression and consistent expression trends were selected as biomarkers.

#### 2.2.6. Gene Set Enrichment Analysis (GSEA)

To assess the relationships between key genes and all genes, Spearman correlation analysis was carried out while employing the psych in R, with criteria of |*R*| > 0.3 and *p* < 0.05. GSEA was conducted based on the correlation rankings using the clusterProfiler in R, and the background gene was utilized to block the signaling pathways potentially involved in the biomarkers.

#### 2.2.7. Immunoinfiltration Analysis

The proportions of immune cell types were compared between the IS and control groups with CIBERSORT. Principal component analysis (PCA) clustering of immune cells was performed using the ggplot2 package, and a stacked histogram was generated. The relationships between immune cells were evaluated via the Spearman correlation analysis, and the correlation heat map was visualized with the help of the corrplot package (https://github.com/taiyun/corrplot). The *t*‐test was carried out for the differential analysis of each immune cell type between patients and controls (*p* < 0.05). Violin plots of the results were compiled using ggplot2 in R.

#### 2.2.8. Drug Prediction

This study also predicted targeted drugs for the biomarkers using the DSigDB database (https://dsigdb.tanlab.org/DSigDBv1.0/). The following drug predictions are computational inferences based on the DSigDB database and should be interpreted as hypothesis‐generating rather than as validated therapeutic targets. Moreover, the interactions between biomarkers and drugs were also assessed. The threshold *p* < 0.05 was considered for selection, and the visualization of the results was carried out using Cytoscape.

### 2.3. Animal Experiments

#### 2.3.1. The Establishment of MCAO/R Model

Male Sprague‐Dawley rats, aged 9–11 weeks and weighing between 200 and 260 g, were acquired from the Experimental Animal Center of Nanjing University. Animals were randomly assigned to sham and MCAO/R groups. The investigator was blinded to group allocation during outcome assessment. The MCAO/R used a nylon filament to induce focal cerebral ischemia [12]. Briefly, rats were anesthetized, secured in a stereotaxic frame, and an incision was made in the midline of the neck. Then, the subcutaneous tissue and muscle were incised, and a 4 cm nylon filament (*ϕ* 0.26–0.28 mm, Xinongkeji, China) was carefully inserted into the middle cerebral artery and kept in place for 120 min. Subsequently, the nylon filament was removed to ensure blood returned to the ischemic artery, and the incision was stitched. The same procedure, but without occusion of the middle cerebral artery, was applied to the Sham group.

#### 2.3.2. Real‐Time Quantitative Polymerase Chain Reaction (RT‐qPCR) Validation

After 24 h of reperfusion, rats were anesthetized and decapitated to harvest their brains. Total RNA was extracted from the penumbra area in the cerebral cortex for qRT‐PCR, which followed a previously defined protocol [13]. The primers used for qRT‐PCR were obtained from Sangon Biotech Co. Ltd. (Shanghai, China), and their sequences are as follows: CDH7‐F: AAGTTACTGCGTGCTGTGAT, CDH7‐R: CTGGAGTGGGCTGAGATTGG, MGAT4C‐F: GCCTTGTGACTGGCTATTGA, MGAT4C‐R: GAGACTTGCTGGTGGGTTAT, ITPKC‐F: GACCCAGAACGCAGCCATAT, ITPKC‐R: TCCTCCCTTGCCTCAGAAAT, GAPDH‐F: AGACAGCCGCATCTTCTTGT, GAPDH‐R: CTTGCCGTGGGTAGAGTCAT. Using the 2^-*ΔΔ*Ct^ method, relative mRNA levels were measured and normalized to GAPDH expression.

#### 2.3.3. Immunohistochemical Staining of Cerebral Cortex

The tissue sections were fixed using paraffin, dehydrated with a serial alcohol solution, closed with 5% goat serum for 1 h, and stained overnight with CDH7 (Servicebio, GB112601, 1:500), MGAT4C (Proteintech, 17841‐1‐AP, 1:100) and ITPKC (Bioss, bs‐18183R, 1:100) primary antibodies at 4°C. Incubation of sections with the corresponding secondary antibodies was performed at ambient temperature for 1 h. This was followed by DAB staining according to the kit′s guide. Hematoxylin counterstaining was performed, followed by imaging under a light microscope. The positively stained areas were then analyzed using ImageJ.

#### 2.3.4. Statistical Analysis

All data were analyzed in R (V4.2.2). For data processing and image modification, online platforms and databases, as well as ImageJ and GraphPad Prism (V7.0) software, were employed. Gene expression differences were analyzed and verified in experimental animal models. The results are given as mean ± SEM. To account for multiple comparisons in the MR analysis of 3589 protein exposures, a Bonferroni‐corrected significance threshold was applied. The threshold for statistical significance was set at *p* < 1.393 × 10^−5^ (0.05/3589). Associations with *p* values below this threshold were considered statistically significant, whereas those with *p* values between this threshold and 0.05 were considered suggestive. Intergroup comparisons were conducted using a two‐sample independent *t*‐test for normally distributed data and Wilcoxon tests for non‐normally distributed data. Statistical significance was considered at *p* < 0.05 unless otherwise specified.

## 3. Results

### 3.1. Selection of SNPs and Genetic Associations of Circulating Proteins with IS

After searching exposure factors and outcome variables and selecting IVs, 3589 exposure factors related to circulating proteins were identified, comprising 13,842 SNPs (Table S1). MR analysis using the inverse‐variance weighted (IVW) method was performed on these 3589 circulating protein‐related exposure factors, identifying 174 proteins with suggestive genetic associations with IS (IVW *p* < 0.05). Detailed IVW effect estimates are provided in Table S2. For the three candidate biomarkers, the IVW method yielded the following effect estimates: CDH7 (OR = 1.18, 95% CI: 1.06–1.31, *p* = 0.003), MGAT4C (OR = 1.24, 95% CI: 1.09–1.41, *p* = 0.001), and ITPKC (OR = 0.79, 95% CI: 0.68–0.92, *p* = 0.002), indicating that genetically predicted higher levels of CDH7 and MGAT4C were associated with increased IS risk, whereas higher ITPKC levels were associated with reduced risk. Table S3 lists the top 10 SNPs independently associated with each circulating protein exposure factor. This study only presents the analysis process of KCNAB2.

**Figure 1:**
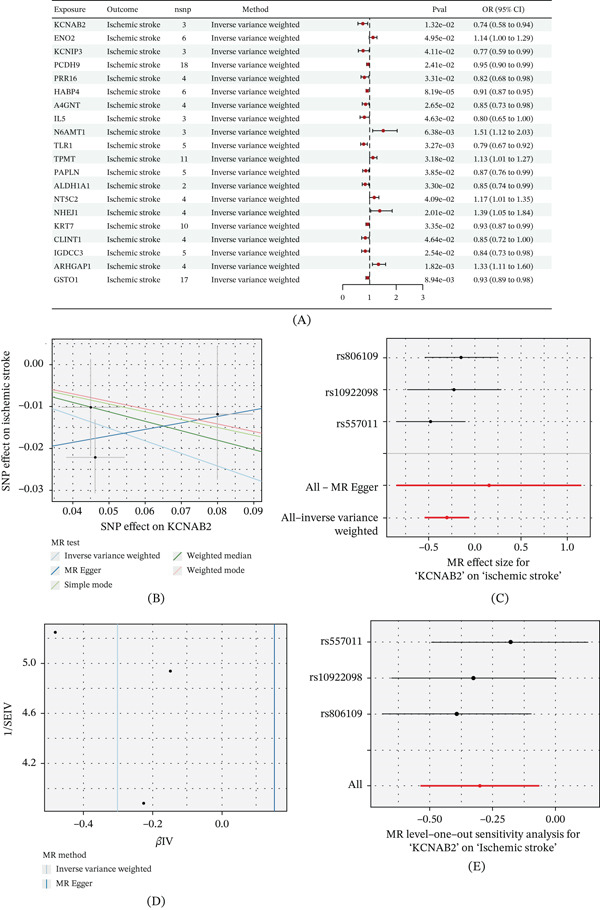
The genetic association of circulating proteins with IS. (A) Forest plot. It illustrates the integrated estimates of SNPs, with the red dots indicating values derived from the IVW method and the horizontal lines denoting 95% confidence intervals. (B) Scatter plot. The slope of the lines in the plot corresponds to the estimated effects assessed by the MR method. (C) Diagnostic efficacy forest plot depicting exposure factors for outcome. (D) Funnel plot. All SNPs estimates are represented by the vertical lines. The funnel plot symmetry indicates no obvious horizontal pleiotropy. (E) Leave‐one‐out analysis. The causal effect, assessed by the IVW method after excluding a particular variant, is represented by the black dots, whereas the red dot shows the IVW estimate based on all SNPs. Abbreviations: IS, ischemic stroke; IVW, inverse‐variance weighted; MR, mendelian randomization; SNP, single‐nucleotide polymorphism.

In total, 171 genes indicating a suggestive genetic association with IS were identified from the 3589 exposure factors for circulating proteins after filtering (Figure 1A). The correlation analysis between the exposure factors and outcomes revealed a negative slope in the IVW analysis, suggesting that the KCNAB2 gene may act as a protective factor. Furthermore, the overall slope was small, indicating a weak effect (Figure 1B). The effect of exposure factors on the outcome was estimated by combining the SNPs (Figure 1C). It was observed that the effect estimates confidence intervals of SNPs (rs806109 and rs10922098) were > 0, indicating that these SNPs may not significantly affect the outcome. However, the effect estimate confidence interval for SNP (rs557011) was < 0, indicating its significant negative effect on the outcome (IS) and suggesting that KCNAB2′s increased exposure may have a protective effect on IS through this SNP.

### 3.2. MR Result′s Validation by Sensitivity Analyses

The MR analysis complied with Mendel′s second law (Figure 1D). The robustness of the MR results was further supported by multiple MR methods (MR Egger, weighted median, simple mode, and weighted mode), which yielded effect estimates consistent with the IVW method for the top 10 exposures (Table S4). The horizontal pleiotropy test indicated *p* > 0.05 for all genes (Table S5), which suggests the absence of horizontal pleiotropy and confounding factors. After filtering, 158 genes met the criteria (Table S3). Furthermore, in the heterogeneity test, 157 genes were selected after excluding variables with Q < 0.05 (Table S6). Moreover, the LOO analysis was performed by sequentially removing each IV to assess its effect on the outcome. The removal of each SNP from KCNAB2 negatively affected the overall size of the IS, suggesting that KCNAB2 may have a protective role (Figure 1E). The directional accuracy of the associations between circulating proteins and IS was confirmed by Steiger′s directionality test (Table S7).

Based on the above results, we identified 157 genes from the MR analysis that exhibit a suggestive genetic association with IS, referred to as MRGs.

### 3.3. Identification of Circulating Proteins Related DEGs and Enrichment Analysis of Candidate Genes in IS

**Figure 2:**
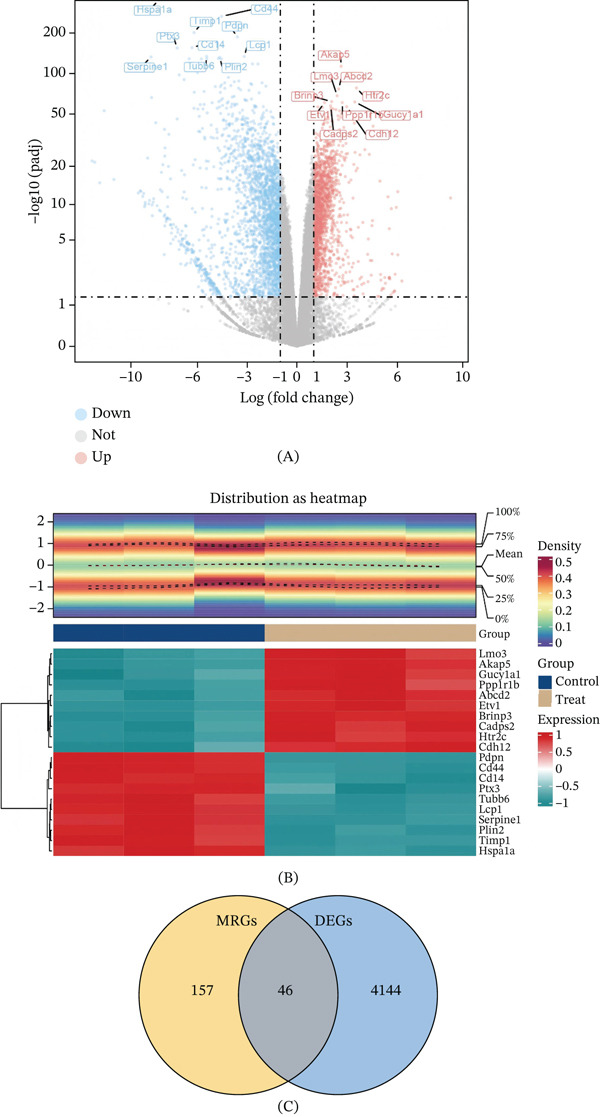
Identification of DEGs and candidate circulating proteins‐related genes. (A) Volcano plot. Upregulated genes are shown as red points, and downregulated genes as blue points, whereas grey points represent genes with no significant change. Differential expression was considered significant with |log2 fold change| > 1 and an adjusted *p* value < 0.05. (B) Heatmap. This heat map displays the top 10 upregulated and 10 downregulated genes, where blue indicates lower expression levels and red indicates higher expression levels. (C) Venn diagram. The DEGs and MRGs showed an overlap of 46 genes. Abbreviations: CSF, cerebrospinal fluid; DEGs, differentially expressed genes; MRG: MR analysis genes.

Batch effects were removed, and the microarray datasets GSE268634 and GSE262257 were merged and differentially analyzed. The data revealed 4144 DEGs, with 2493 downregulated, and 1651 upregulated DEGs in the disease group. Then, the volcano plot of all DEGs and the heat map of the top 10 upregulated genes and the top 10 downregulated genes were visualized (Figure 2A,B). The analyses identified 46 genes overlapping in MRGs and DEGs. These genes were regarded as candidate genes linked with IS (Figure 2C).

The biological pathways and functions of 46 candidate genes were assessed via the enrichment analysis. GO analysis showed that candidate genes were primarily enriched in BP, such as cell‐substrate adhesion, cell‐matrix adhesion, negative regulation of fibrinolysis, collagen‐containing extracellular matrix, external side of the plasma membrane, endoplasmic reticulum lumen, endopeptidase regulator activity, and integrin binding (Figure S2A). Furthermore, KEGG analysis further highlighted their involvement of candidate genes in Th17 cell differentiation, Toll‐like receptor, and RIG‐I‐like receptor pathways (Figure S2B), suggesting that the immune regulation pathway was associated with IS pathogenesis.

The PPI network investigated the molecular mechanisms in physiological and pathological processes and indicated 10 nodes and 22 edges (Figure S3A). TLR1 was identified as the core node in the network with the most connections. CD86 and CSF3 also indicated numerous connections.

### 3.4. CDH7, MGAT4C, and ITPKC Are Dependable Biomarkers

To further filter candidate biomarkers for IS, the LASSO algorithm was applied to identify 16 key genes from 46 candidate genes (Figure S3B,C). Furthermore, the SVM‐RFE algorithm (Figure S3D,E) identified 20 key genes. The intersection of these 16 and 20 genes (Figure S3F) revealed 6 key genes.

**Figure 3:**
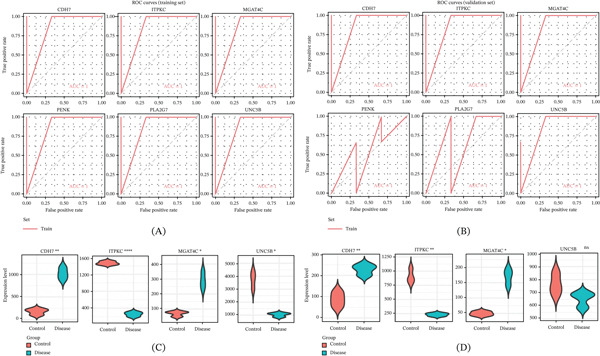
Identification of biomarkers. (A and B) The candidate biomarkers were assessed through ROC curve analysis in both the training and validation sets. (C and D) Candidate biomarker expression levels are displayed for both the training and validation sets. Genes with AUC > 0.7 in both training and validation datasets and consistent differential expression (CDH7, MGAT4C, and ITPKC) were selected as final putative biomarkers and are highlighted with asterisks (∗) in panels C and D. ∗*p* < 0.05, ∗∗*p* < 0.01, ∗∗∗*p* < 0.001, ∗∗∗∗*p* < 0.0001, Abbreviation: ns, not statistically significant.

The ROC curves were used to evaluate the effectiveness of the supervised machine learning algorithms. ROC curve analysis of the six key genes showed that CDH7, ITPKC, MGAT4C, and UNC5B had good predictive performance and AUC > 0.7 in both the training and validation datasets, making them potential candidate biomarkers (Figure 3A,B). The expression levels of key genes were further validated in IS and healthy control groups (Figure 3C,D), which showed that CDH7, MGAT4C, and ITPKC were significantly differentially expressed in both datasets, with consistent expression trends. Therefore, they are proposed as putative biomarkers for IS.

### 3.5. GSEA of Biomarkers

**Figure 4:**
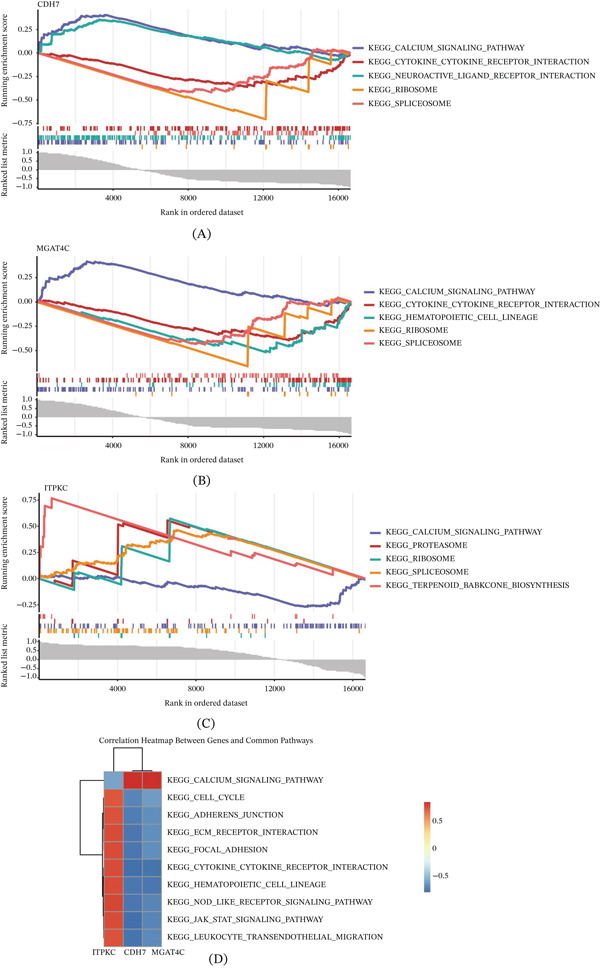
GSEA analysis of biomarkers. Top 5 GSEA enrichments identified in genes with high and low expression levels including (A) CDH7, (B) MGAT4C, (C) ITPKC. and (D) Correlation analysis between biomarkers and pathways.

GSEA identified the biological pathways enriched by the three biomarkers based on their expression values (Figure 4A–C). It was observed that CDH7 was positively correlated with the calcium signaling pathway and neuroactive ligand‐receptor interaction signaling pathway, whereas it was negatively correlated with the cytokine‐cytokine receptor interaction, ribosome, and spliceosome pathways. MGAT4C indicated a positive correlation with the calcium signaling pathway and negative correlations with the cytokine‐cytokine receptor interaction, hematopoietic cell lineage, protein synthesis (ribosome), and RNA splicing (spliceosome) pathways. The proteasome, ribosome, spliceosome, and terpenoid backbone biosynthesis were positively correlated with ITPKC, whereas the calcium signaling pathway was negatively correlated with it. Furthermore, the correlation analysis of biomarkers and the enriched pathways demonstrated that CDH7 and MGAT4C were positively correlated with the calcium signaling pathway but negatively correlated with other pathways. Whereas ITPKC indicated an opposite trend (Figure 4D).

### 3.6. The Immune Infiltration Was Markedly Variable Between the IS and Control Groups

**Figure 5:**
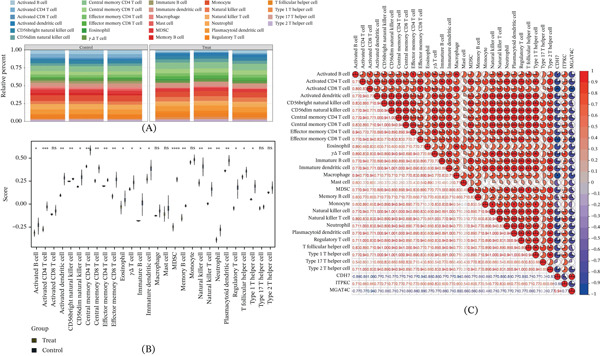
Distribution of immune cells in IS. Immune infiltration analysis. (A) The proportion of immune cells in the IS and control groups is shown in a stacked bar plot. (B) Differences in 28 immune cell types between the IS and control groups are depicted in the violin plot. (C) Correlation heat map between immune cells and biomarkers. The following symbols were employed for indicating the significance levels: ∗*p* < 0.05, ∗∗*p* < 0.01, ∗∗∗*p* < 0.001, ∗∗∗∗*p* < 0.0001, Abbreviations: IS, ischemic stroke; ns, not statistically significant.

As shown in Figure 5A, the distribution of different immune cell types is compared between the IS and control groups, along with the outcomes of the reverse analysis. The differential analysis in the enrichment scores of 28 immune cell types in the IS and healthy groups revealed that 22 immune cells had significantly different infiltration levels (*p* < 0.05) (Figure 5B). Compared to the control group, the IS group indicated a downward trend in the relative expression of activated B cells and activated CD4 T cells, whereas natural killer and Type 1 T helper cells indicated increased relative abundance. Further correlation analysis showed that ITPKC had a positive association with immune cells, but CDH7 and MGAT4C had negative correlations (Figure 5C).

### 3.7. Validation of Biomarkers Expression in MACO/R Rats and Drug Prediction for IS Therapy

**Figure 6:**
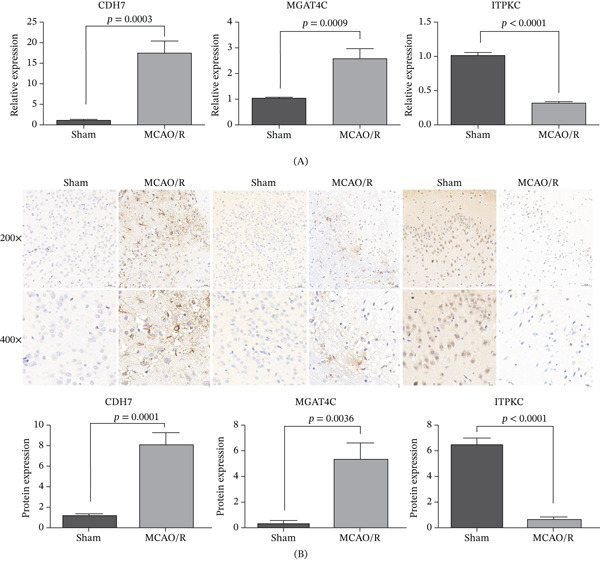
Validation of the expression of identified biomarkers in MCAO/R rats. (A) The relative expression of these biomarkers was evaluated via RT‐qPCR. (B) The expression of biomarkers in the cortex was detected by immunohistochemistry and, and ImageJ analyzed the average optical density values. n = 3 biological replicates per group.

RT‐qPCR analysis of the three biomarkers was performed, which revealed that the mRNA expression of CDH7 and MGAT4C was significantly high in the cerebral cortex, whereas that of ITPKC was markedly reduced (Figure 6A). Moreover, the results of immunohistochemistry experiments further validated qRT‐PCR data and indicated increased CDH7 and MGAT4C protein expression while reduced ITPKC protein expression in the cerebral cortex (Figure 6B).

At last, we also predicted targeted drugs for CDH7, MGAT4C, and ITPKC using the DSigDB database and visualized the results using Cytoscape (*p* < 0.05) (Figure S4). The following drug predictions are computational inferences based on the DSigDB database and should be interpreted as hypothesis‐generating rather than as validated therapeutic targets. Experimental validation is required to confirm any direct interactions or therapeutic efficacy. The PPI network comprised 18 nodes and 15 edges. Genistein, pioglitazone, rosiglitazone, and troglitazone indicated neuroprotective effects by alleviating oxidative stress and inflammation, thereby mitigating ischemic reperfusion brain injury, but direct evidence of targeting CDH7, MGAT4C, or ITPKC is currently lacking.

## 4. Discussion

IS is a leading cause of morbidity and mortality worldwide [14], yet effective biomarkers for early diagnosis and targeted treatment remain limited. Our study used MR to identify proteins genetically associated with IS risk, overlapped these with DEGs from an IS rat model, and validated their dysregulation in vivo. Although our MR analysis was based on plasma protein GWAS data, the rationale stems from their putative relevance to CSF, as discussed in the limitations. These converging lines of evidence suggest CDH7, MGAT4C, and ITPKC are promising candidates for further functional investigation.

From the MR analysis, 3589 circulating protein‐related exposure factors were examined, resulting in the identification of 171 genes with suggestive causal relationships to IS. After rigorous sensitivity tests‐including pleiotropy, heterogeneity, and LOO analyses, 157 genes were retained as MRGs, providing a robust foundation for downstream investigation. Among these, regulatory beta subunit 2 of voltage‐gated potassium channel (KCNAB2) [15] showed a weak yet potentially protective effect on IS, particularly through SNP rs557011, suggesting variants in circulating protein levels can influence stroke susceptibility.

To further refine candidate biomarkers, differential gene expression analysis was performed on merged microarray datasets. A total of 4144 DEGs were identified, of which 46 overlapped with the MRGs. These overlapping genes represent strong candidates with both causal evidence and altered expression in IS, providing a valuable subset for further functional investigation.

Functional enrichment analysis revealed that the 46 candidate genes were predominantly involved in pathways associated with extracellular matrix interaction, immune modulation, and signal transduction. GO and KEGG pathway analysis indicated significant involvement in cell adhesion, fibrinolysis regulations, and immune‐related pathways such as toll‐like receptor and Th17 cell differentiation. These findings align with established IS pathophysiology, where inflammation, immune cell infiltration, and extracellular matrix remodeling play critical roles in neuronal damage and recovery [16, 17].

To identify the most robust biomarkers, machine learning algorithms (LASSO and SVM‐RFE) were applied, ultimately narrowing the list to six key genes. ROC curve analyses demonstrated that CDH7, MGAT4C, and ITPKC had consistent diagnostic accuracy with AUC values above 0.7 across both training and validation datasets. These genes were selected for further characterization as putative biomarkers for IS.

CDH7, encoding cadherin‐7, is involved in calcium‐dependent cell adhesion and neurodevelopment processes [18]. Its upregulation in IS suggests a potential role in mediating neural cell‐cell interactions during injury and repair. MGAT4C, a glycosyltransferase, influences glycoprotein synthesis and immune cell signaling [19]. Its elevated expression in IS may contribute to post‐ischemic immune activation or neuroinflammation. ITPKC, in contrast, was downregulated in IS and is known to regulate calcium signaling [20] and T cell activation [21], suggesting a protective immunomodulatory role.

GSEA further supported these roles. CDH7 and MGAT4C were positively associated with the calcium signaling pathway but negatively associated with inflammatory and protein synthesis pathways, potentially reflecting their involvement in excitotoxicity and neuronal remodeling. In contrast, ITPKC was negatively correlated with calcium signaling and positively correlated with pathways related to protein degradation and biosynthesis, indicating a distinct role in limiting inflammatory responses and restoring cellular homeostasis.

Immune infiltration analysis revealed significant alterations in immune cell profiles in IS patients, with 22 immune cell types showing differential abundance compared to controls. Notably, activated B cells and CD4+ T cells were reduced in IS, whereas pro‐inflammatory cell types such as natural killer and Th1 cells were elevated, suggesting a significant role of inflammation in IS development [22]. Correlation analyses showed that ITPKC expression was positively associated with beneficial immune cells, whereas CDH7 and MGAT4C were negatively associated, reinforcing the idea that ITPKC may act as an anti‐inflammatory factor.

Finally, hypothesis‐generating drug prediction analysis identified several small molecules‐such as genistein [23] and pioglitazone [24] that may target these biomarkers and offer neuroprotective benefits by reducing oxidative stress and inflammation. However, direct evidence linking these compounds to CDH7, MGAT4C, or ITPKC is currently lacking, and experimental validation is required. Experimental validation in an MCAO/R rat model confirmed the differential expression of CDH7, MGAT4C, and ITPKC, consistent with the computational findings and supporting their translational potential.

This study presents several limitations. The primary one is its reliance on bioinformatic analysis conducted using data from a public database, and the clinical effect of these genes needs further experimentation. Second, this study selected young animal models, which may restrict the practical application of the findings to the elderly population affected by IS. Third, this study used cross‐species validation (human genetic data vs. rat models). Despite high gene homology, species‐specific differences may exist; however, the consistency of expression trends across species strengthens biological plausibility. Fourth, direct evidence from human CSF samples is lacking. Although circulating proteins can reflect CNS processes, future studies should validate their levels in paired CSF and plasma from IS patients. The term “CSF” here refers only to future validation, not to the current exposure data. Fifth, the drug prediction analysis is hypothesis‐generating and requires experimental confirmation. Future prospective human validation is needed to bridge this gap.

## 5. Conclusions

In conclusion, our integrative analysis identifies CDH7, MGAT4C, and ITPKC as genetically associated and transcriptionally altered candidates derived from circulating protein analyses for IS. These findings suggest that these genes may serve as potential biomarkers and warrant further functional investigation to elucidate their mechanistic roles in IS pathogenesis.

**Nomenclature**
AUC: area under the curve
BP: biological processes
CC: cellular components
CSF: cerebrospinal fluid
DEGs: differentially expressed genes
GEO: Gene Expression Omnibus
GSEA: gene set enrichment analysis
GWAS: genome‐wide association studies
IS: ischemic stroke
IVs: instrumental variables
IVW: inverse‐variance weighting
LASSO: least absolute shrinkage and selection operator
LD: linkage disequilibrium
LOO: leave‐one‐out
MF: molecular functions
MR: mendelian randomization
OR: odds ratios
PCA: principal component analysis
PPI: protein‐protein interaction
ROC: receiver operating characteristic
SNPs: single nucleotide polymorphisms
SVM‐RFE: support vector machine‐recursive feature elimination

## Author Contributions

Jingwei Xiong: conceptualization, formal analysis, and writing – original draft; Jie Zhang: data curation and methodology; Xuemei Cheng: investigation and validation; Zhiqiang Zhou: software and visualization; Lidong Zhang: supervision and writing – review and editing; Chunlong Chen: project administration and writing – review and editing. Lidong Zhang and Chunlong Chen contributed equally as co‐corresponding authors.

## Funding

No funding was received for this manuscript.

## Disclosure

All authors read and approved the submitted manuscript.

## Ethics Statement

All animal experiments were approved by the Ethics Committee of Jinling Hospital (Approval No. DZGZRDW2400173) and complied with ARRIVE guidelines.

## Consent

The authors have nothing to report.

## Conflicts of Interest

The authors declare no conflicts of interest.

## Supporting Information

Additional supporting information can be found online in the Supporting Information section.

## Supporting information


**Supporting Information 1** Figure S1 The flowchart of the study design.

**Supporting Information 2** Figure S2 Enrichment analysis of GO and KEGG pathways related to 46 candidate genes. (A) The top 10 GO terms in the biological process (BP), cellular component (CC), and molecular function (MF) categories. (B) KEGG pathway analysis of candidate genes.

**Supporting Information 3** Figure S3 PPI analysis and machine learning analysis. (A) The PPI network of top 10 molecules. (B,C) Candidate biomarkers screening *via* the LASSO model and (D,E) the SVM‐RFE model. (F) Overlapping genes identified by the two algorithms were screened using a Venn diagram. PPI: Protein‐protein interaction.

**Supporting Information 4** Figure S4 Drug‐target interaction network for CDH7, MGAT4C, and ITPKC. The network was constructed using the DSigDB database (*p* < 0.05) and visualized with Cytoscape. Circles represent drugs, diamonds represent target genes, node colors indicate drug classes (e.g., isoflavones, thiazolidinediones), and edges represent significant associations.

**Supporting Information 5** Table S1: Instrumental variables (SNPs) for each circulating protein exposure. Table S2: Odds ratios and effect sizes for key Mendelian randomization findings. Table S3: Information on SNPs independently associated with circulating protein exposure factors (top 10). Table S4: Evaluation of MR analysis results for valid IVs for circulating protein exposures using multiple methods (top 10). Table S5: Horizontal pleiotropy test (top 10). Table S6: Heterogeneity test analysis (top 10). Table S7: Steiger directionality test (top 10). STROBE‐MR checklist of recommended items to address in reports of mendelian randomization studies.

## Data Availability

The datasets generated for this study can be found in Mendeley Data via doi:10.17632/ms5pcfsm9x.1.
