# Supplementary figures and images for: Mendelian Randomization and Transcriptome Analysis Identify Ischemic Stroke Biomarkers With Putative Relevance to Cerebrospinal Fluid
Source: Biomed Res Int. 2026 Jul 8;2026:2880611. doi: 10.1155/bmri/2880611 (PMC13343476; doi:10.1155/bmri/2880611)

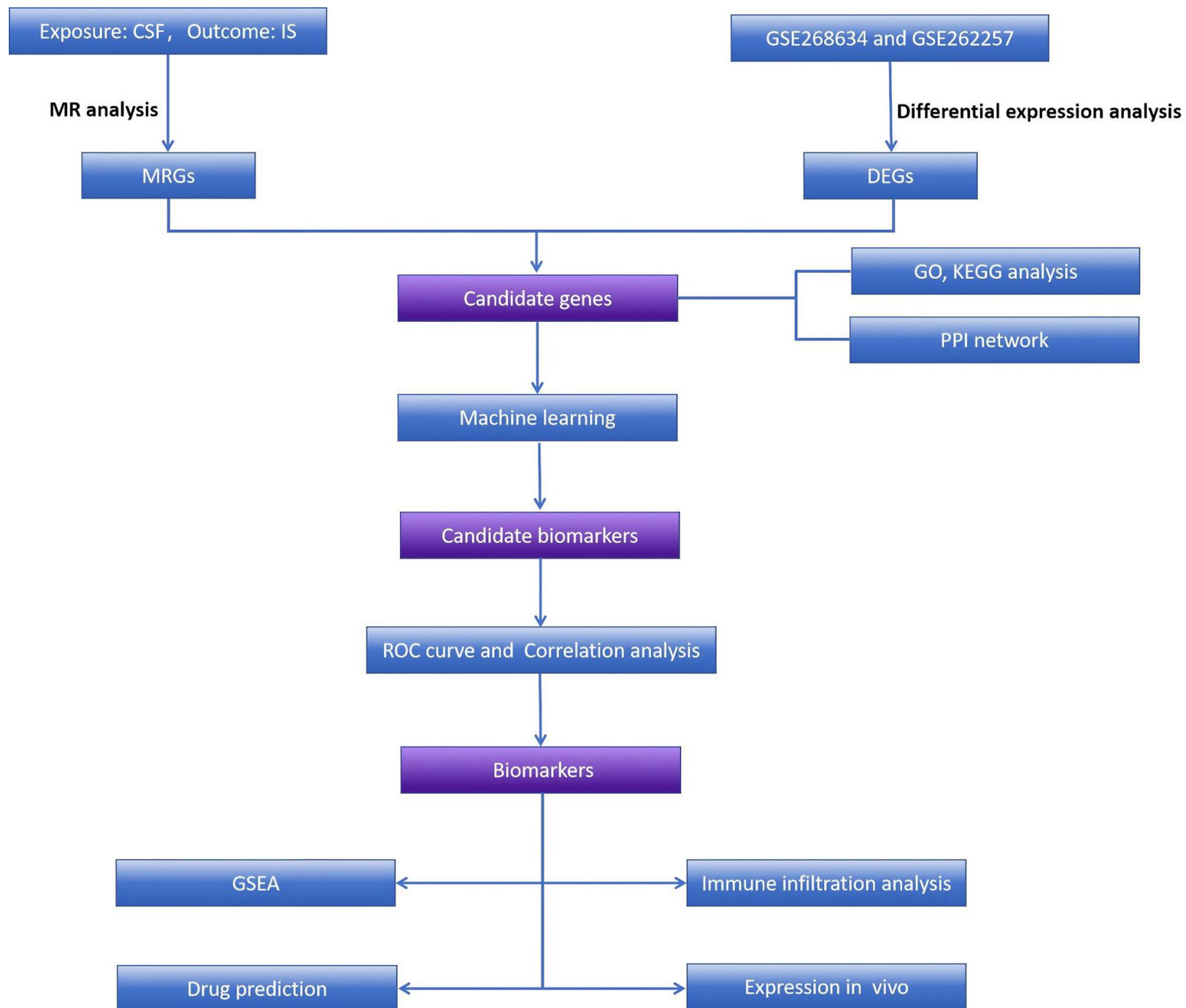

Supplement: Supplementary file 1 — Supporting Information 1 Figure S1 The flowchart of the study design. [file BMRI-2026-2880611-s004.pdf]

**A**

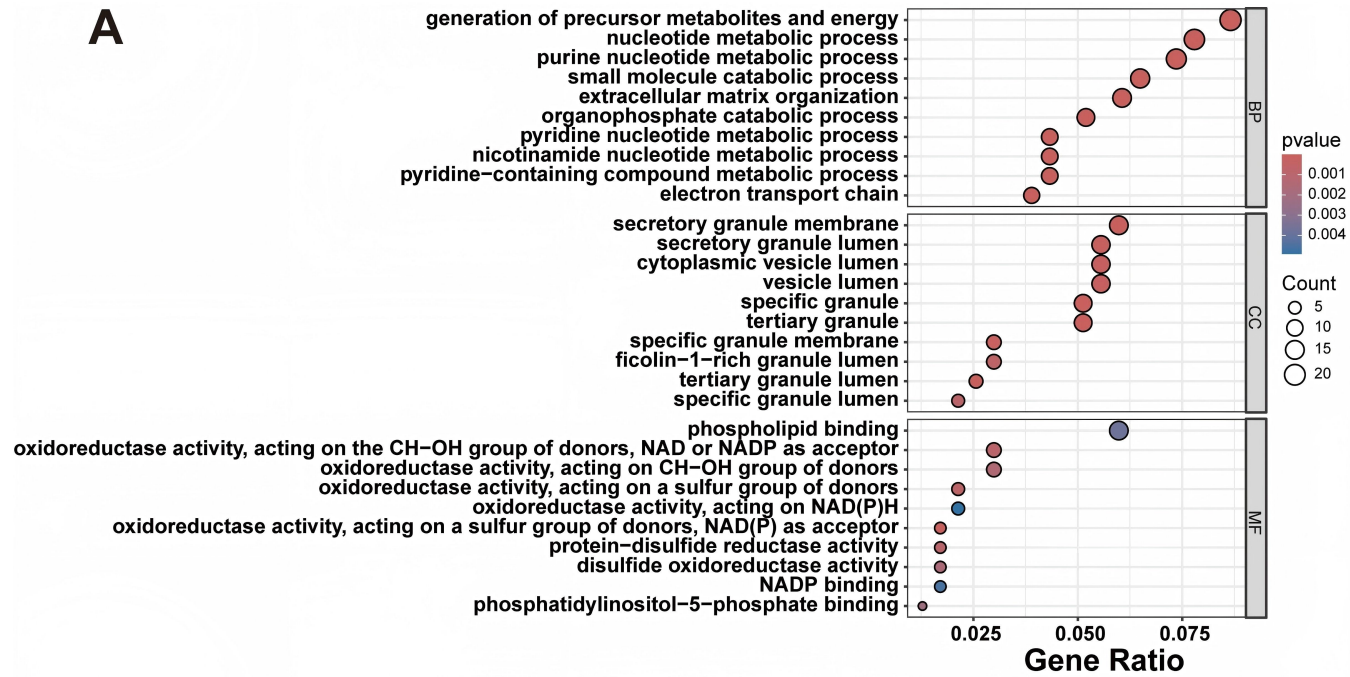

**B**

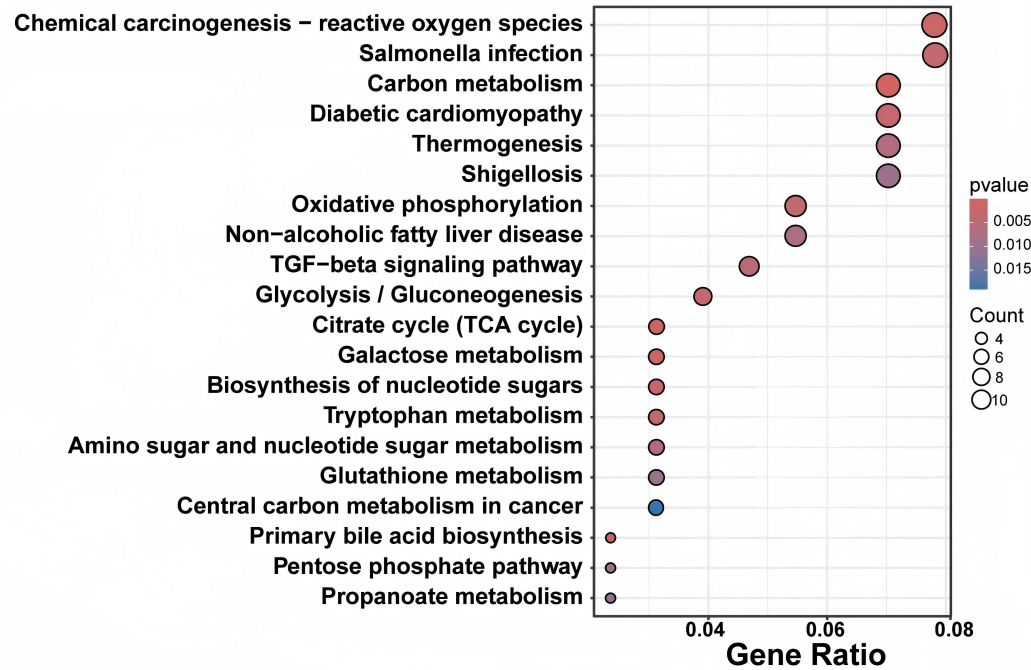

Supplement: Supplementary file 2 — Supporting Information 2 Figure S2 Enrichment analysis of GO and KEGG pathways related to 46 candidate genes. (A) The top 10 GO terms in the biological process (BP), cellular component (CC), and molecular function (MF) categories. (B) KEGG pathway analysis of candidate genes. [file BMRI-2026-2880611-s003.pdf]

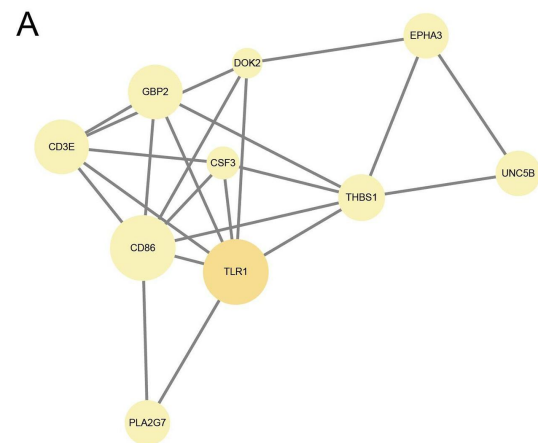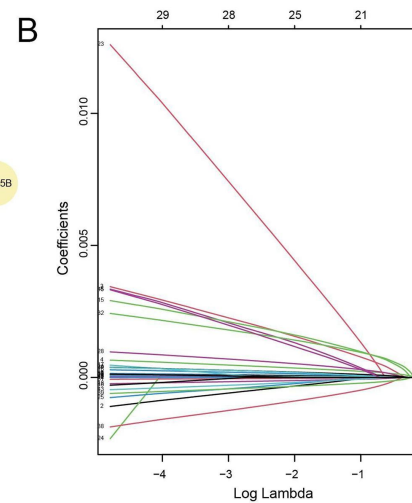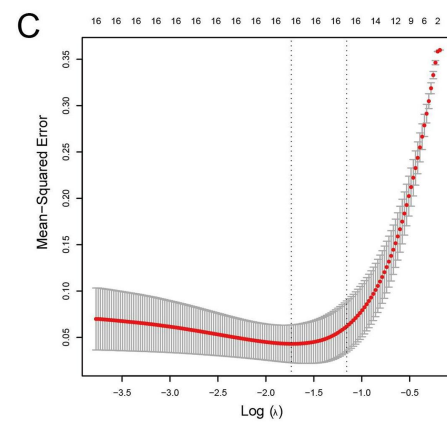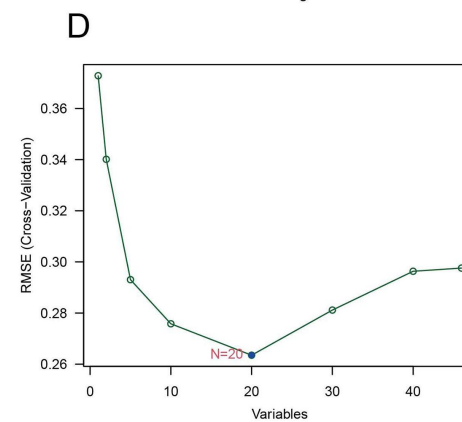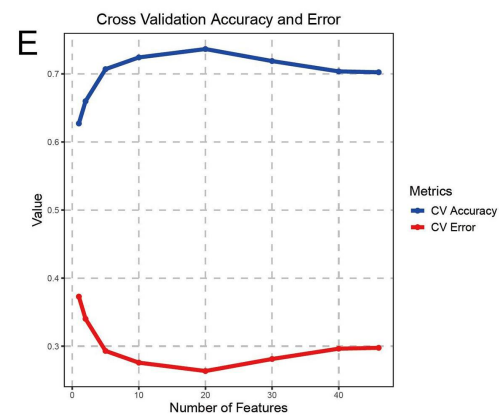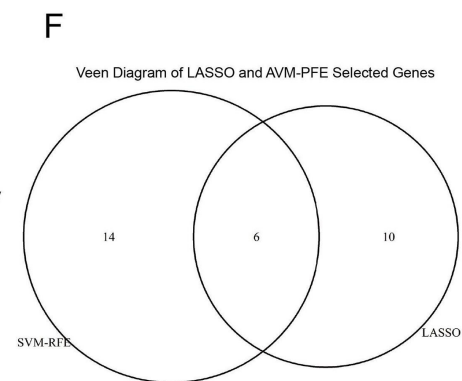

Supplement: Supplementary file 3 — Supporting Information 3 Figure S3 PPI analysis and machine learning analysis. (A) The PPI network of top 10 molecules. (B,C) Candidate biomarkers screening via the LASSO model and (D,E) the SVM‐RFE model. (F) Overlapping genes identified by the two algorithms were screened using a Venn diagram. PPI: Protein‐protein interaction. [file BMRI-2026-2880611-s002.pdf]

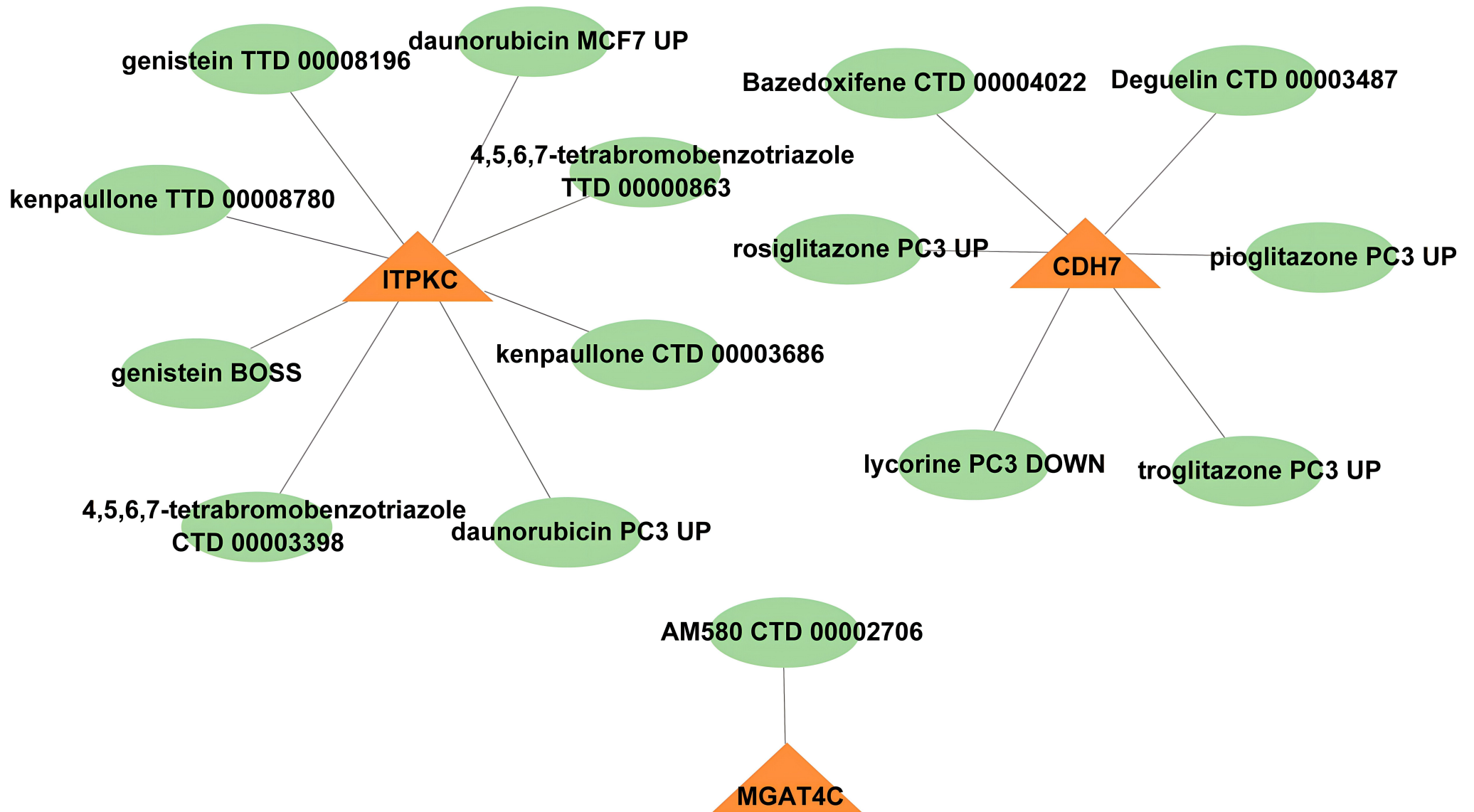

Supplement: Supplementary file 4 — Supporting Information 4 Figure S4 Drug‐target interaction network for CDH7, MGAT4C, and ITPKC. The network was constructed using the DSigDB database (p < 0.05) and visualized with Cytoscape. Circles represent drugs, diamonds represent target genes, node colors indicate drug classes (e.g., isoflavones, thiazolidinediones), and edges represent significant associations. [file BMRI-2026-2880611-s001.pdf]
